# Model-Based Reinforcement of Kinect Depth Data for Human Motion Capture Applications

**DOI:** 10.3390/s130708835

**Published:** 2013-07-10

**Authors:** Luis Vicente Calderita, Juan Pedro Bandera, Pablo Bustos, Andreas Skiadopoulos

**Affiliations:** 1 Polythecnic School of Cáceres, University of Extremadura, Avd. de la Universidad, Cáceres 10003, Spain; E-Mail: pbustos@unex.es; 2 Department of Electronic Technology, University of Málaga, Campus de Teatinos, Málaga 29071, Spain; E-Mail: jpbandera@uma.es; 3 Biomechanics of Human Movement and Ergonomics Lab, BioẼrgon Research Group, University of Extremadura, Avd. de la Universidad, Cáceres 10003, Spain; E-Mail: andreas@unex.es

**Keywords:** human motion capture, sensor, RGB-D sensors, range camera, pose analysis

## Abstract

Motion capture systems have recently experienced a strong evolution. New cheap depth sensors and open source frameworks, such as OpenNI, allow for perceiving human motion on-line without using invasive systems. However, these proposals do not evaluate the validity of the obtained poses. This paper addresses this issue using a model-based pose generator to complement the OpenNI human tracker. The proposed system enforces kinematics constraints, eliminates odd poses and filters sensor noise, while learning the real dimensions of the performer's body. The system is composed by a PrimeSense sensor, an OpenNI tracker and a kinematics-based filter and has been extensively tested. Experiments show that the proposed system improves pure OpenNI results at a very low computational cost.

## 1. Introduction

There are many application fields for systems able to capture human motion. These fields include, but are not limited to medical and rehabilitation scenarios, human-machine interfaces, computer graphics animation applied to video games or movies, surveillance, service robotics, *etc*. Traditional human motion capture (HMC) systems rely on a set of markers worn by the human performer to capture her movements. These systems are expensive, have to be employed in controlled environments, usually require extensive calibration processes and are invasive for the person [1,2]. Besides, the data provided by these systems often require additional filtering and alignment processing stages, which complicate their use in *on-line* applications.

Different alternatives to these approaches have been investigated in the last few years with the aim of overcoming the limitations of marker-based systems. These initiatives look for a low-cost system able to capture motion *on-line* and to achieve markerless HMC in uncontrolled environments. Despite the considerable efforts performed, these approaches have achieved a limited degree of success [1], in part due to some specific issues, which are listed below.

- *Person detection*. It is complicated to perceive objects and people in a noisy, uncontrolled environment. Object detection algorithms based on stereo vision are affected by lighting variations, shadows and untextured surfaces or objects. More sophisticated range sensors, such as infrared-based depth sensors, are still affected by partial perception and occlusions. Person detection algorithms have not only to deal with these issues, but they also have to discriminate among people and other objects.
- *Body parts identification*. In order to capture the motion of a person, it is necessary to identify her body parts. This is a complex task if it has to be achieved without markers, special garments or clothes.
- *Motion tracking*. HMC systems aim not only at perceiving static poses, but tracking the motion of a human performer. Natural movements can involve fast motions that exceed the capture rate of most vision-based systems. Tracking losses become a common issue for these systems.
- *Speed*. In order to avoid the previous issue and to provide a more detailed description of the performed motion, commercial marker-based HMC systems are able to capture human motion at high frame rates, which may exceed 60 frames per second (fps). However, they require post-processing stages to refine results and filter outliers. On-line HMC systems have to address the problem of providing a robust estimation of the motion at a high enough frame rate. This requirement constrains the complexity of the algorithms used by such systems.

In the last year, the field of markerless, unconstrained HMC has experienced an important advance, due to the appearance of several cheap RGB-D sensors and new fast and robust pose estimation algorithms. Among these systems, the combination of the PrimeSense sensor and the OpenNI open source framework is one of the most common options. The sensor, used in the Kinect commercial device, is cheap, but nevertheless provides a quite accurate depth map of the environment [3]. The NITE middleware in the OpenNI framework allows one to track human motion from these depth data at a high frame rate (up to 30 fps). They overcome most previous approaches in terms of robustness, speed and accuracy.

While the benefits of this system are clear, it does not guarantee the validity of the provided pose. Thus, limb lengths may change drastically (e.g., an object in the hand will be considered as a *part* of the arm), joint angle limits are not evaluated (e.g., an elbow could bend in both directions), self-collisions are not checked and pose ambiguities are not solved.

**Figure 1.**
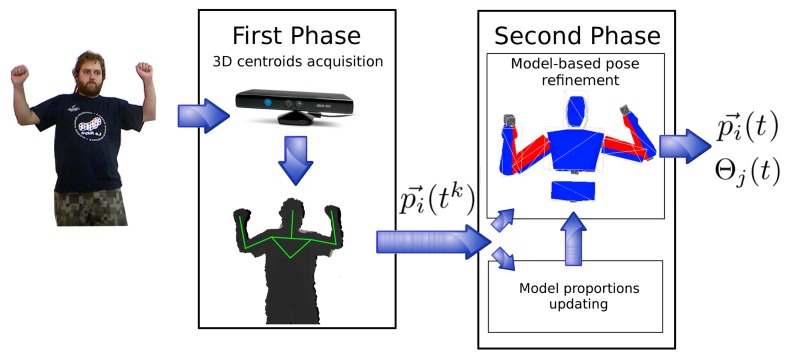
System overview.

In this paper, a system is proposed that addresses this issue using a kinematics model of a human to enforce and guarantee the validity of obtained poses. In doing so, it tracks human motion in two steps, as depicted in Figure 1. The first step collects 3D centroids of different body parts using the OpenNI HMC algorithm; the second step uses a model-based filter to estimate a valid human pose from these intermediate data. Collected input data are also used to adapt the used model to the particular characteristics of the human being tracked. Both procedures (pose checking and adaptation) are executed in parallel (Figure 1) and do not reduce the frame rate of the original HMC system provided by OpenNI framework.

Our HMC system has been quantitatively evaluated through two sets of experiments. Experiments in the first set measure limb lengths and instant position errors for different arm gestures, using a commercial, multi-view marker-based system as the ground-truth. The second set of experiments evaluate the percentage of correctly estimated joint positions in a set of labeled motion sequences. The results obtained are deeply discussed in the following sections.

The rest of the paper is organized as follows: Section 2 describes related work to help understand the scope and characteristics of the proposed approach. Section 3 details the model-based kinematics filter used to extract a valid human pose from OpenNI data. The algorithms used to learn the proportions of the performer are detailed in Section 4. Section 5 evaluates the obtained experimental results. Finally, Section 6 discusses the advantages and drawbacks of the proposed method and explores its application fields and future improvements.

## 2. Related Work

Non-invasive approaches that estimate and track the configuration of a complex, articulated object can be divided in two main categories [4,5]: (i) *learning-based* or *model-free* approaches, which rely on probabilistic and search methods to infer the human pose from image cues; and (ii) *model-based* approaches, which use a model of the perceived person to compute her pose.

### 2.1. Model-Free Approaches

These approaches directly map perception to pose space. They rely on extensive databases composed of sets of perceived cues associated with poses. Capturing human motion using these approaches becomes a matter of whether a problem is of performing search and match operations in these databases or of off-line learning of a classifier function binding perception and pose spaces. These processes may be time-consuming and have to deal with the problems of local minima or silhouette ambiguities [4,6]. Generalization from the set of demonstrated poses may be also difficult if this set is not complete enough [7]. Despite these issues, many researchers have used these approaches, due to their ability to extract poses from cluttered natural scenes and single views [1].

Model-free approaches have experienced a recent growing interest since the publication of the Shotton *et al*. contributions [8]. These contributions describe the method Microsoft uses in its Kinect for Windows SDKto extract the human pose, from the depth data provided by the PrimeSense sensor. Its main characteristics are the following: (i) it uses depth images, instead of color images, to infer human poses; (ii) it uses a decision forest to classify features extracted from the depth image; and (iii) the decision trees are trained using hundreds of thousands of poses, performed by virtual models and recorded using virtual RGB-D cameras. A color texture is applied to the virtual models that helps in identifying body parts in the images (color and depth pixels match exactly, as a virtual, ideal camera is being used to record the poses). The trained system is able to extract 3D centroids of different body parts using mean-shift over per-pixel density estimators, which weight each pixel considering both the inferred body part probability at the pixel and the world surface area of the pixel. The resulting 3D centroids are located on the surface of the body, and they are pushed back in the scene by a *z* learned offset [8].

The main characteristics of the method of Shotton *et al*. are its robustness and speed. It is able to capture full-body motion at 30 fps in uncontrolled environments without imposing any requirement on the performer. It is also able to provide coherent estimations for occluded joints [8]. Its main drawbacks are its difficulties in inferring a correct pose for the human legs, its limitations in capturing fine motion (e.g., finger motion) and its absence of a kinematics coherence in the provided poses. Thus, the algorithm of Shotton *et al*. provides only estimated 3D positions of different body parts, but it does not check limb lengths, joint limits nor collisions [8]. The OpenNI framework also includes an HMC algorithm in its NITE middleware. To the best of our knowledge, the NITE algorithm has not been published, but its functionality and performance approach the ones of the algorithm of Shotton *et al*. It also shares its issues regarding occlusions and the absence of kinematics coherence [9].

### 2.2. Model-Based Approaches

Model-based approaches rely on the use of a human model to help in the person detection and tracking processes. Traditionally, these approaches offered faster results than model-free solutions, as they do not need to perform search operations in large databases. They are also more robust against ambiguities, incorrect poses and self-occlusions, as the model helps in inferring a valid pose for a given perceived data and provides pose estimations for occluded joints [10,11]. Finally, they do not need to rely on extensive training phases, as information about the human kinematics and dynamics can be stored in the model itself [1,4]. Among model-based approaches, the use of explicit kinematics, shape and appearance models in analysis-by-synthesis frameworks are the most common option [1,12]. Other approaches include creating new models from observations [13].

While these approaches are adequate in terms of motion tracking and speed, they face issues regarding person and body parts identification. Model-fitting algorithms are required to start the capture process. These algorithms are bounded by the detail level of the model used. Rough models are usually easier to match in these initialization processes, and they reduce the computational load in the tracking process. However, they are less precise in their estimations. On the other hand, there are parameters of the model (*i.e.*, lengths and widths) that should be adapted to the particular performer to correctly capture her motion. Some contributions assume models with fixed dimensions, but this strategy leads to inaccurate pose estimations [5]. Other approaches use an initialization stage [11] or rely on statistical priors [14]. Zhang *et al*. propose to use manual calibration or model shape fitting to perform this adaptation [9]. On-line adjustment of these parameters has been addressed, but using multiple cameras [15].

These issues related to person identification and model initialization do not exclude the application of model-based criteria to modify the estimations provided by an existing human tracking process. Besides, systems that use a model to refine perceived poses may benefit from using complex, realistic models, as these models do not need to be included in computationally expensive (usually recursive) model-fitting processes. Thus, recent contributions combine model-free (bottom-up) and model-based (top-down) approaches to extract human joint angles from perceived cues [9,16]. However, the use of a model does not only help in solving pose ambiguities or obtaining joint angles. As pointed out by Poppe [5] in his survey about vision-based HMC, model-based approaches can also apply constraints to prune the pose space and eliminate infeasible poses. These constraints include joint angle limits [11,17], velocity and acceleration limits [18] and collision constraints [11]. While these techniques have been extensively used in the last decade in vision-based HMC applications [5], to the best of our knowledge, they have only been marginally used with PrimeSense sensor. This paper helps in analyzing whether these complex models are useful to reinforce HMC systems based on the PrimeSense sensor. It presents a two-step HMC algorithm, in which the first step provides a rough pose estimation, while the second step refines this estimation using model-based criteria. More precisely, the paper describes a system that uses a kinematics human model to improve HMC data provided by the PrimeSense sensor and OpenNI framework.

## 3. A Human Kinematics Filter

**Figure 2.**
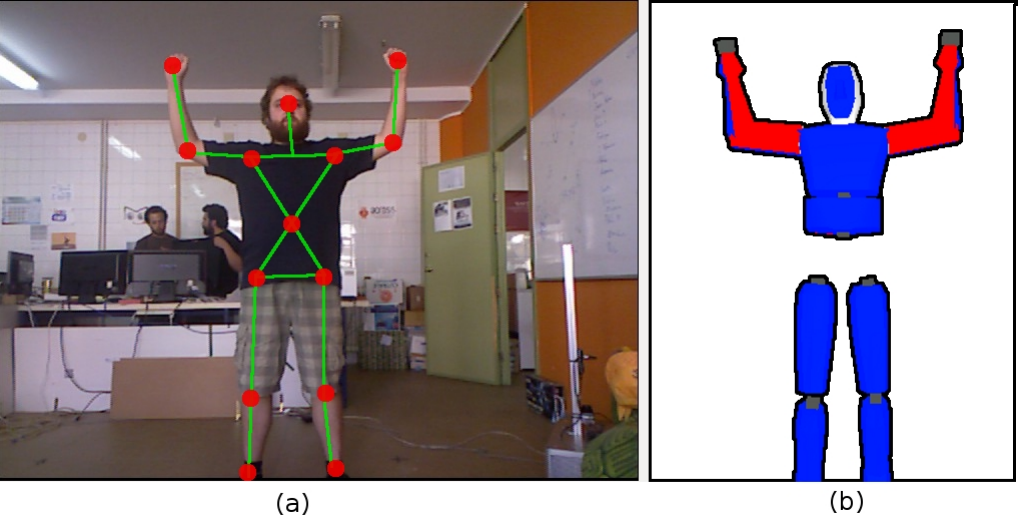
**(a)** 3D centroids of body parts provided by OpenNI tracker (red dots); and (**b**) kinematics model employed to encode and refine perceived human pose.

**Figure 3.**
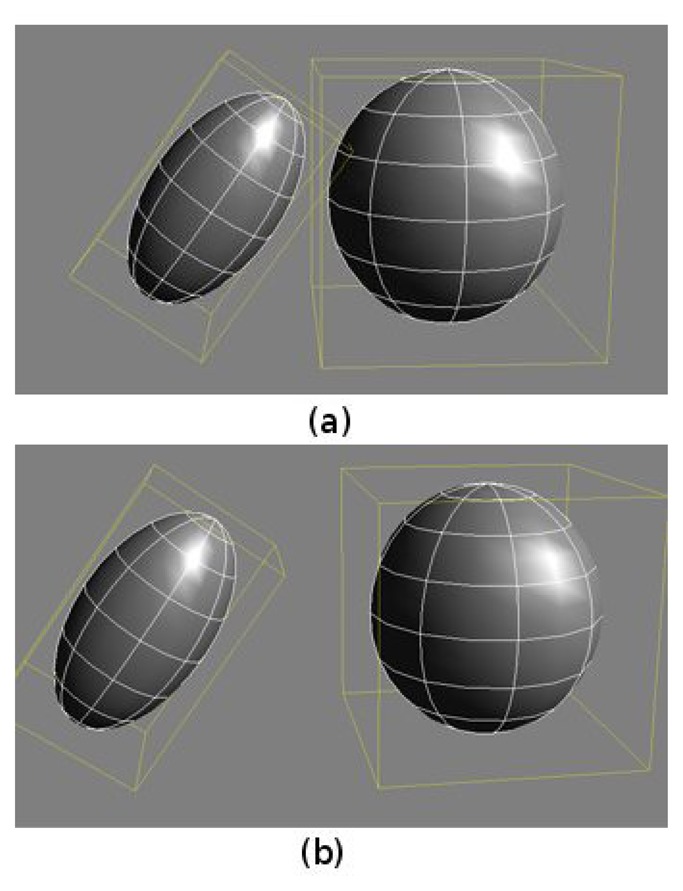
An example of the two-level collision detection algorithm.

The OpenNI tracker provides an estimation of 3D centroids of different body parts (Figure 2a). These 3D centroids obtained by the OpenNI framework do not necessarily satisfy human kinematics, which promptly produces several issues when used to estimate the pose of a person. The use of surface depth data to compute 3D joint positions may also lead to errors when estimating the pose for occluded joints. This paper proposes to include a second step in the HMC process to address these limitations. This second step uses the 3D centroids provided by the OpenNI framework to feed a model-based pose generator. The core of this pose generator is a human model, depicted in Figure 2b. This model includes:

- Kinematics constraints. Each joint contains data about allowed degrees of freedom (DOF) and joint angle limits. These limits are set according to the maximum values provided by anthropometric tables [19].
- Self-collision avoidance algorithms. The method used in this paper follows an efficient two-step collision detection process, which can be considered a simple example of a multiresolution static collision detection algorithm [20]. In this case, only two levels of detail are used: bounding box level and detailed mesh level. Thus, the method executes a first collision detection process at the bounding box level and a second detection process at the mesh level, only if bounding boxes are colliding. This approach increases the speed of the algorithm, especially if complex, realistic meshes are used. Figure 3 shows an example of the use of the two-step collision detection algorithm: the bounding boxes of the objects in Figure 3a collide; thus, the collision detection at the mesh level has to be performed. On the other hand, the bounding boxes of the objects in Figure 3b are not colliding; thus, this second step is not executed, significantly increasing the speed of the detector.
- The capability of modifying its limb dimensions on-line.

These capabilities of the model allow one to detect invalid poses. These poses are avoided using an alternative evaluation algorithm that was previously contributed [2]. This algorithm is based on the analytic inverse kinematics (IK) solver proposed by Mitchelson [21] to obtain joint angles, given the shoulder and hand 3D positions. The algorithm analyses the output of this IK solver and checks its validity. Invalid poses are replaced by alternative poses that fit the constraints of the kinematics model. The end effector position is located as close as possible to the desired position for these alternatives (see Chapter 3 in [2] for a detailed explanation about the complete algorithm).

Finally, the capability of the model to modify its limb dimensions will allow it to adapt to each performer, as further detailed in Section 4.

**Figure 4.**
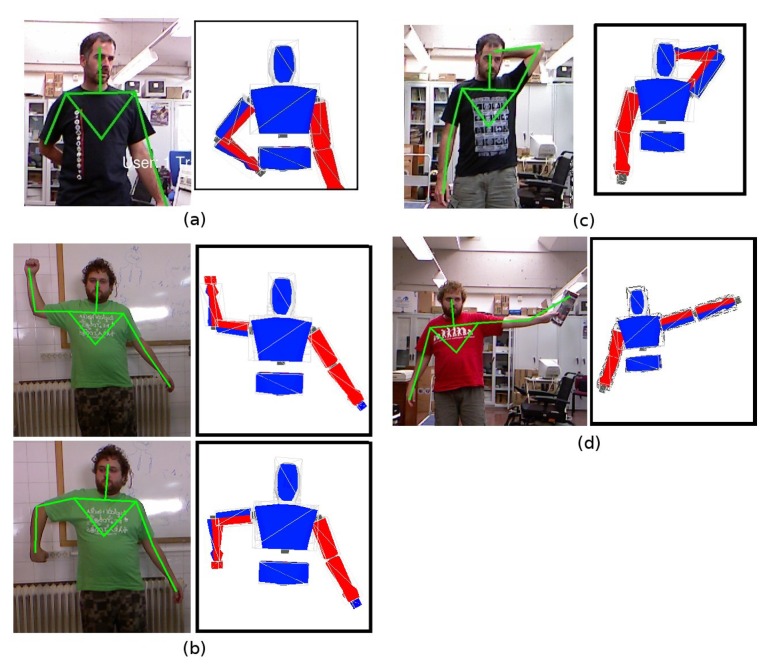
Examples of OpenNI issues and corresponding model poses.

Figure 4 highlights several issues of the OpenNI tracker and how the proposed model-based HMC system allows one to provide a valid human pose despite them. The first example frame, depicted in Figure 4a, shows an occlusion of the right hand 3D centroid. The OpenNI tracker does not provide reliable position data for the hand, nor for the elbow (whose 3D position is provided, but with a lower associated confidence value). The model uses position data obtained for the previous poses to infer a forearm pose that, as Figure 4a depicts, approaches the pose of the performer.

Joint angles can be extracted from the 3D positions provided by the OpenNI tracker. There are several applications and published examples in which the transformation from 3D positions to joint angles is performed. The proposed system, however, is also able to avoid odd poses and guarantee a valid angle configuration for every frame. Figure 4b depicts a situation in which the model adopts a pose that matches 3D centroids provided by the OpenNI tracker and satisfies human motion constraints. Figure 4b shows that the model arm always bends at a valid angle (model limbs have been colored in blue and red to better show this characteristic of the model).

The pose depicted in Figure 4c is an example in which OpenNI tracker fails in providing a correct 3D centroid for the left hand. The performer poses his hand behind his head. However, the OpenNI tracker does not consider the hand centroid lost, as most of the forearm is visible. Thus, it takes depth values for the hand centroid from the depth map. These depth values do not always correspond to hand depth, but for some frames, head depth values are taken instead. The proposed HMC method avoids the collision between the head and the hand for these frames. Thus, the model maintains the hand behind the head.

Finally, Figure 4d depicts a situation in which the OpenNI tracker provides an incorrect 3D centroid for the left hand, due to the presence of an object. This error propagates towards the model, which tries to locate its hand in the erroneous position. However, the knowledge the model incorporates allows it to recognize that the OpenNI centroid is unreachable. The model stretches its arm completely, but keeps it kinematically coherent.

Let *L* be the number of 3D centroids provided by the OpenNI tracker (*L* = 8 for upper-body HMC). Let *EE* ⊂ [0..*L*] be the subset of end-effectors (e.g., hands) and *IJ* ⊂ [0..*L*], the subset of intermediate joints (e.g., elbows). Let $\vec{p_{i}}(t^{k})=(x_{i}(t^{k}),y_{i}(t^{k}),z_{i}(t^{k}))$, *i* ∈ [0..*L*] be the 3D positions of centroids provided by the OpenNI tracker, referring to the person's torso, *O* = (*x_o_*, *y_o_*, *z_o_*), at time instant, *t^k^*. Let ${\vec{p_{i}}}^{m}(t^{k})=(x_{i}^{m}(t^{k}),y_{i}^{m}(t^{k}),z_{i}^{m}(t^{k}))$, *i* ∈ [0..*L*] be the 3D positions of human model joints, referring to the person's torso, at time instant, *t^k^*. The model-based pose generator algorithm searches for the best valid pose given the available observations provided by the OpenNI tracker. This process is described by Algorithm 1:

**Algorithm 1** Human pose estimation.
**Require:** centroids1:**for each**
*centroid* ∈ *centroids***do**2: replace by value in previous frame if low confidence value3: if *end effector*, compute valid position using IK4: if not *end effector*, correct centroid value5: move corrected centroid to validCentroids6:**end for**7:**for**
*t* ∈ (*t^k^*, *t^k^*^+1^) **do**8: add new spline interpolated centroid at *t*9: self-collision checking of interpolated centroid using IK at *t*10: add centroid to validCentroids11:**end for**12:**return** validCentroids


Each step of the algorithm is further explained below.

- The OpenNI tracker provides a confidence value, *c*(*i^k^*), for each 3D centroid. Unreliable data have a confidence value of zero, estimated data for occluded centroids have a confidence value of 0.5, while correctly perceived centroids have a confidence value of one. As Section 5 discusses, pose estimations provided by OpenNI for occluded centroids may lead to overlapping limbs and incorrect poses, but the model avoids these issues. Thus, in line 2, the algorithm replaces unreliable 3D centroids using Equation (1). Only centroids that have not been estimated by PrimeSense sensor and OpenNI tracker are not updated.
  (1) $$\begin{array}{l} \forall i\in EE\;/c(i^{k})<0.5\;\Rightarrow \vec{p_{i}}(t^{k})=\vec{p_{i}}(t^{k-1})\\ \forall i\in IJ\;/c(i^{k})<0.5\;\Rightarrow \vec{p_{i}}(t^{k})={\vec{p_{i}}}^{m}(t^{k-1}) \end{array}$$
- In line 3, the end-effector positions of the model for time instant, *t^k^*, are computed using Equation (2):
  (2) $$\forall i\in EE\;{\vec{p_{i}}}^{m}(t^{k})=IK(\vec{p_{i}}(t^{k}),\vec{p_{j}}(t^{k})/j\in K_{i})$$
  where *K_i_* is the set of joints included in the kinematics chain that connects *O* and joint *i* and *IK* () is an implementation of the inverse kinematics (IK) algorithm detailed in [2]. This algorithm solves for end effector and intermediate joints given the root of the chain (*i.e.*, the shoulder) and the end effector position. The algorithm is fast, as it relies on analytic relations to solve the IK problem. It also includes mechanisms to avoid self-collisions and joint limit violations. Finally, it guarantees that the provided 3D positions, ${\vec{p_{i}}}^{m}(t^{k})$, correspond to a valid human pose.
- In line 3, the human model adopts the pose that locates its end-effectors in the 3D positions computed in the previous step. The 3D positions of the intermediate joints, ${\vec{p_{i}}}^{m}(t^{k})/i\in IJ$, are obtained by reading their values from the model.
- Once all the valid 3D centroids, ${\vec{p_{i}}}^{m}(t^{k})$, *i* ∈ [0..*L*], have been obtained, a third order spline interpolator is used in line 8 to create smooth trajectories, ${\vec{p_{i}}}^{i}(t)$, *i* ∈ [0..*L*], that go from the pose the model had at time instant, *t^k^*^−1^, to the new desired pose at time instant, *t^k^*. The use of spline interpolators to generate natural motion from sets of key-points is a common practice in computer graphics and animation or robotics research fields [22–24]. This paper follows the same criteria and considers obtained centroids as the key-points for the spline interpolator. As further explained in Section 5, the use of an interpolator will also help in matching perceived trajectories against the ones captured using a different HMC system. On the other hand, a third order interpolator needs both the positions of the 3D centroids and their trajectory slopes at time instant, *t^k^*^−1^. Thus, the interpolator introduces a constant delay of two samples. None of the users who have tested the system for gesture imitation applications has reported any issue related to this delay. However, for some applications (e.g., video games) an average delay of ∼66 msec—which corresponds to two frames captured at 30 fps—may be significative. The third order interpolator can be replaced by a linear interpolator [2] or even discarded if a faster response time is required.
- While the validity of the model poses in the sample instants, *t^k^* and *t^k^*^−1^, is guaranteed, it may be possible to go through invalid poses in the interpolated trajectory that goes from one pose to the other (e.g., the two arms may collide). In line 9, the IK algorithm is used again to check each new interpolated pose. Therefore, the trajectories followed by the human model end-effectors are computed as ${\vec{p_{i}}}^{m}(t)=IK({\vec{p_{i}}}^{i}(t),{\vec{p_{j}}}^{i}(t)/j\in K_{i})$, ∀*i* ∈ *EE*.

## 4. Learning the Dimensions of the Human Figure

The pose estimation algorithm performs two simultaneous tasks, as depicted in Figure 1: (i) it estimates human poses using a human model; and (ii) it adapts the dimensions of the model according to the particular person being tracked. This second task is achieved by using an adaptive filter, which takes as input the set of 3D centroids provided by the OpenNI tracker, $\vec{p_{i}}(t^{k})$. To adapt the human model to the actual person dimensions, we divide the HMC process in three phases, *initialization, learning and fixation*.

### Initialization

The learning procedure is initialized using the anthropometric relations published in the works of Contini [19]. These data have been collected over a wide variety of live people and provide the average proportions of mature male and female humans, relative to *H* (distance from the floor to the top of the head).

### Learning

*H* is computed, according to the method of Contini, as *H_e_*/0.936, *H_e_* being the floor-to-eye distance. However, while eye positions are successfully detected by the algorithms of the Microsoft Kinect SDK, the different versions of the OpenNI framework do not always perceive eyes. Thus, in this paper, *H_e_* is estimated as the average face centroid extracted from OpenNI data. If the person stands still during the initialization phase, the height coordinate for this face centroid approaches the value of *H_e_*. Once the model height has been updated, its limb lengths, $\bar{l_{j}}(t^{k})$, are also adapted to the perceived human data only during the first *N* samples using an *alpha-beta* filter with time-varying coefficients (Algorithm 2).



**Algorithm 2** Learning human limb dimensions.
**Require:**
*Limbs, CorrectedLimbs, k* ∈ *Frames* Λ (*k* > 0)1:
$\alpha =\frac{k-1}{k}$, 
$\beta =\frac{1}{k}$2:**for each**
*l* ∈ *Limbs*, *l̅* ∈ *CorrectedLimbs***do**3: **if** (*k* < *N*) ⋁ (|*l̅* − *l*| > *MaxDist*) **then**4:  *l̅_t_*_+1_ ← *α* * *l̅_t_* + *β* * *l_t_*5: **end if**6:**end for**7:**return**
*CorrectedLimbs*
which can also be re-framed as a cumulative running average algorithm:

(3) $$\forall k\in [0..N]\;\bar{l_{j}}(t^{k})=\bar{l_{j}}(t^{k-1})+\frac{l_{j}(t^{k})-\bar{l_{j}}(t^{k-1})}{k}$$

where *l_j_*(*t^k^*) is the instant limb length for frame *k*, computed as the distance between the 3D centroids of the ending joints (the frames in which OpenNI does not provide a reliable centroid are discarded). After *N* samples, the measures are considered stable, and learning freezes, unless a significative disparity between the model and the data is discovered.

### Fixation

After the first *N* samples, the model proportions are still updated using Algorithm 2. If the distance between the learned length and the computed length grows over a certain threshold, *MaxDist*, the length of that limb is re-evaluated by setting the *k* value to zero and performing the learning phase again, for that limb.

## 5. Results and Discussion

The proposed two-step HMC algorithm has been evaluated through two extensive quantitative evaluation processes.

The first of these processes evaluates instantaneous position errors for free arm motion sequences. Hands and elbows are clearly visible for the OpenNI tracker (OpenNI 1.5.4 and NITE 1.5.2.21 were used in all the experiments) in these sequences. Three sets of values are considered for each test: (i) the set of 3D centroids provided by the OpenNI tracker; (ii) the final poses estimated by the human model if limb lengths are not updated; and (iii) the final poses estimated by the human model when the learning algorithm introduced in Section 4 is activated. These tests are used to analyze the validity of the proposed algorithm against a reliable ground-truth and the effects of the limb adaptation algorithm in the accuracy of the provided pose.

The proposed algorithm is specially designed to help correctly capture human motion when the OpenNI tracker may fail. Thus, the second group of experiments considers four motion sequences in which occlusions and overlapping are present. These sequences are divided into regions defined by the relative positions of hands and elbows with respect to the head or torso. Six different people of different gender, age and proportions performed the sequences, providing approximately half an hour of motion data. The evaluation of these data compares the percentage of correctly detected frames for both the proposed algorithm and the OpenNI tracker. It also analyzes the accuracy of the limb length learning algorithm for different performers.

### 5.1. Experiment 1. Instantaneous Position Errors Evaluation

The following paragraphs describe the set-up, data preprocessing stages and obtained results for the first set of experiments.

#### 5.1.1. Experimental Set-Up

The motion of the human performer has been recorded for these experiments using two different HMC systems: (i) the HMC system proposed in this paper; and (ii) a MaxPRO (Innovision Systems, Inc. [25]) on-line data acquisition and a software device that incorporates a motion capture (MoCap) system with six infrared (IR) cameras working at 30 to 120 fps with a maximum camera resolution of 659 × 494 pixels and a pixel size of 5.6 × 5.6 *μ*m. This second HMC system has been taken as the ground-truth; thus, the instant position errors for each joint can be obtained as the Euclidean distance between the 3D positions provided by both systems.

**Figure 5.**
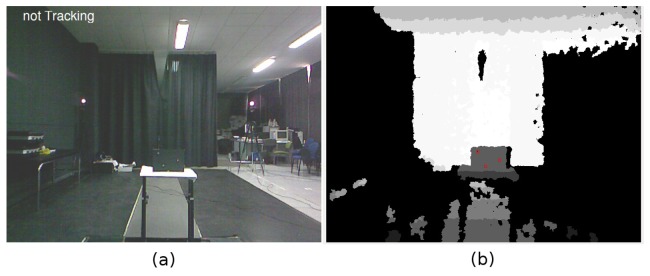
**(a)** Capture volume for the quantitative evaluation of the system; and (**b**) selection of the three markers in the calibration plane.

The experiments have been conducted in a specific scenario, the Biomechanics of Human Movement and Ergonomics Lab (University of Extremadura), due to the requirements of the MaxPRO commercial system. This scenario includes six IR cameras located around the human performer to minimize the effects of occlusions. These cameras record the 3D positions of a set of passive markers located in a working volume wide enough to allow for unconstrained human arm motion. These positions are recorded at a sample rate of 60 Hz. The average error of the system is about two millimeters, and therefore, it can be used as a reliable ground-truth. The motion of the human performer has been also captured using our HMC system, composed of a PrimeSense sensor connected to a standard PC. The distance from the performer to the PrimeSense sensor is about 1.70 m, although it varies among different tests. Figure 5a shows the working volume as it is perceived by our HMC system. Two MaxPRO cameras can also be seen in the figure.

After testing several configurations of the experimental set-up involving different numbers of markers, human movements and relative positions of both HMC systems, the final scenario consisted in a human performing a right arm motion wearing six IR markers. The markers are located in pairs—two in the wrist, two in the elbow and two in the shoulder—to facilitate the accurate capture of the joints' 3D positions.

### 5.1.2. Trajectory Alignment

Before the tests can be conducted, it is necessary to calibrate the relative positions of both HMC systems. We have obtained a transformation matrix that translates 3D points between HMCs using three IR markers located on a plane (Figure 5b), plus the IR light spot emitted by the Kinect sensor. The image in Figure 5b shows the plane containing the three markers from the point of view of the PrimeSense sensor. These four points are used by each HMC system to compute the relative pose of the other.

For the commercial HMC system, the 3D coordinates of the three markers along with the infrared light spot (detected as an additional marker) are automatically captured and a coordinate system readily computed. For our HMC system, a simple calibration software has been developed using the OpenNI library. This program allows the user to click on the RGB-D image to capture the 3D coordinates of the markers and sets the Kinect infrared light spot as the origin of this coordinate system. The positions of the IR CMOSsensor and the IR light spot in the Kinect are different; thus, an additional constant transformation is applied to correct the captured positions. Once both coordinate systems have been obtained, the transformation matrix that maps the points from one system to the other can be easily obtained solving the following matrix equation:

(4) $$\underset{RT}{\left[\begin{array}{cc} R & T\\ 0 & 1 \end{array}\right]}\underset{A}{\;\left[\begin{array}{cccc} {\bar{p}}_{1} & {\bar{p}}_{2} & {\bar{p}}_{3} & {\bar{p}}_{4}\\ 1 & 1 & 1 & 1 \end{array}\right]}\;=\;\underset{B}{\left[\begin{array}{cccc} {\bar{q}}_{1} & {\bar{q}}_{2} & {\bar{q}}_{3} & {\bar{q}}_{4}\\ 1 & 1 & 1 & 1 \end{array}\right]}$$

where *R* and *T* are the block components of the transformation matrix we need and *p̅_i_* and *q̅_i_* are the four 3D points captured from each HMC system, respectively Solving for *RT*, we obtain:

(5) $$RT=BA^{-1}$$

as our transformation matrix.

Another problem that has to be tackled is the difference in the sampling rates of both HMC systems. While the commercial system maintains a stable sample frequency of 60 Hz, the sampling rate of our HMC system varies, its average value being 30 Hz with a mean deviation below one frame per second. This effect is mostly due to the non real-time nature of the underlying operating system (Linux) and the implementation of the OpenNI software. In order to compare the 3D trajectories captured by both systems, the trajectories captured using our HMC system have been oversampled using third order spline interpolation to match the sampling rate of the commercial HMC system.

The last issue regarding trajectory comparison is related to synchronization. An external signal has been employed to trigger the capture. While this signal is shared by both HMC systems, the use of non real-time operating systems limits the final synchronization precision that can be achieved using this method. In order to refine the temporal alignment of both sets of trajectories, the following steps have been executed for each captured motion:

- To obtain the temporal deviation values for the hand (*cc_h_*), elbow (*cc_e_*) and shoulder (*cc_s_*). These values are computed as the maximum peaks of the cross-correlation of the joint trajectories recorded by both HMC systems.
- To check which of the three deviations (*cc_h_*, *cc_e_*, *cc_s_*) produces a maximum average correlation when applied simultaneously to hand, elbow and shoulder trajectories.
- To apply the previous temporal deviation to the three trajectories.

Once the trajectories have been aligned, both in space and time, the instantaneous position error can be computed, finally. The results obtained are discussed below.

#### 5.1.3. Limb Length Adaptation

The OpenNI tracker provides 3D centroids, and instantaneous limb lengths can be computed from these 3D centroids; but, these values accumulate important errors, due to incorrect centroid estimations (Figure 4) and perceptual noise. The HMC system presented in this paper obtains stable, robust limb length values from these input data using a running average algorithm to estimate the true proportions of the model.

**Figure 6.**
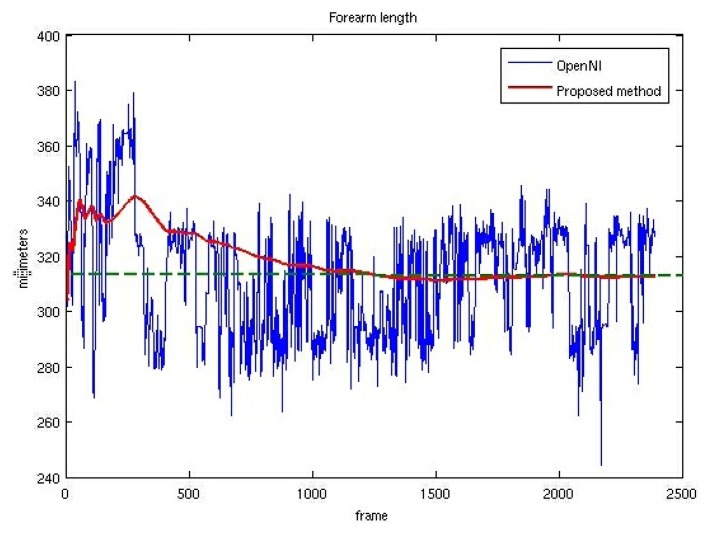
Forearm lengths provided by the OpenNI tracker and by the proposed model-based adaptive system.

Figure 6 shows the instant right forearm lengths computed as the Euclidean distance between 3D centroids provided by OpenNI. It also shows the instant model forearm lengths, computed using the algorithm detailed in Section 2. The measured forearm length (312 mm) is also depicted. Figure 6 shows convincingly how the forearm length of the model converges to the real value despite the noisy values provided by the OpenNI tracker.

The right upper arm length follows a similar variation. The model length converges to 305 mm, the real arm length value being 300 mm. These tests show that the proposed limb length adaptation algorithm provides results accurate enough for being employed in most HMC applications.

#### 5.1.4. Quantitative Error Characterization for Visible Joints

Three experiments have been conducted that involve extensive, free right arm motion; the position of the performer, her orientation, the speed and the characteristics of the motion change among the different tests. The first two tests lasted for 10 s, while the last one has a duration of 30 s.

Table 1 shows the mean errors and standard deviations of the joints tracked in the three tests, for more than 1,500 frames. As commented on above, three sets of values have been measured to show the effects of the model limb length adaptation process.

The shoulder errors are not affected by the use of the model. The tests involve only right hand motion, and the shoulder position is nearly constant in these movements. Its associated error is below 2.5 cm for all tests (Table 1). The hand and the elbow follow more dynamic trajectories. In these tests, the hand position is detected by the OpenNI tracker in all frames (*i.e.*, the confidence value for the wrist 3D centroid is over 0.5 for all frames). Hand joint errors, however, are higher than shoulder joint errors, due to the noise introduced by the hand relative pose in its perceived 3D centroid. The model follows these erroneous positions as they lay in the reachable hand space. Thus, the refined trajectory in these tests is nearly similar to the trajectory of the OpenNI centroid.

The analysis of the elbow joint errors offers different results. The OpenNI tracker provides a value for the elbow 3D centroid for all frames, but it does not check limb lengths coherence nor the validity of the resulting pose. The elbow 3D centroid oscillates around the real elbow, these variations being higher when the human stretches her arm. These issues increase the elbow joint errors for the OpenNI tracker.

The proposed human model, however, constrains the elbow motion to reachable, valid positions. It filters the deviations of the elbow 3D position as model limb lengths converge towards stable values in the learning phase (Section 5.1). Thus, even though the OpenNI tracker provides elbow 3D centroids for all the frames processed in these tests, these values are modified as the model elbow adopts the 3D position that is closest to the centroid provided by OpenNI, but constrained by the human model kinematics. Results depicted in Table 1 show that the proposed method improves the results of the OpenNI tracker, decreasing both elbow mean error and standard deviation.

The validity of the limb length adaptation process is also depicted in Table 1. The second set of values for each joint are obtained for a human model, whose proportions are not adapted from, but take the values provided by, Contini [19]. The shoulder and hand errors are not relevant in this test, as their positions are still fully reachable for the model. However, the elbow errors show that a model with unadjusted limb lengths may produce higher mean position errors than the OpenNI tracker, as the model arm needs to adopt a different configuration to reach the same desired hand position as the human performer. The standard deviation for these errors is still lower than the deviation for the OpenNI tracker, as the model trajectories are smoother, regardless of limb lengths.

The proposed method, which adapts limb lengths to the proportions of the perceived human, improves these results and provides lower errors than both the OpenNI tracker and the model using unadapted limb lengths.

### 5.2. Experiment 2. Pose Estimation for Movements Involving Occluded or Overlapped Joints

The set-up and results obtained in the second set of experiments are detailed below.

#### 5.2.1. Description

The second set of experiments includes upper-body motion sequences that have been captured using both the proposed HMC system and the OpenNI tracker. The PrimeSense sensor is located at a height of 1.50 m. The only requisite for the performers is to stand in front of the sensor, at a distance in which their upper-body motion can be fully captured by the OpenNI tracker.

The experiments involved four different motion sequences (these sequences are available at http://www.grupoisis.uma.es/SequencesSensor.zip). The sequences include gestures in which the hands are occluded behind the head, the torso or the elbows. They also include gestures in which hands and elbows overlap with the head, the torso or other joints (*i.e.*, shoulders, elbows or hands). The four sequences have been performed by six people of different gender, age and proportions. The gestures were briefly described to the performers prior to the execution. This description included a single demonstration for each motion sequence. The performers had to reach relative positions as specified, but no constraints were imposed regarding motion speed nor particular arms or torso poses.

The motion of hands and elbows has been divided for each test into regions. Each region is labeled by the performer, according to the following categories:

- Joint *i* behind the reference joint.
- Joint *i* aligned with the reference joint.
- Joint *i* in front of the reference joint.

where joint *i* can be the right hand, the left hand, the right elbow or the left elbow. The reference joint is the head or the torso, depending on each particular motion sequence. A joint is included in one category or another, for each frame, by comparing its distance to the camera with the distance from the reference joint to the camera and thresholding the result. Table 2 provides a description of the four motion sequences, their reference joints and the employed distance thresholds, Θ*_d_*.

The threshold, Θ*_d_*, varies to match the perception of ‘behind’, ‘aligned’ and ‘in front of’ for each test. Thus, the hands touch the back of the performer in Experiment #3, which uses a Θ*_d_* of 100 mm. However, Experiment #4 uses a bigger Θ*_d_*, as it involves motion in which the arms move close to, but do not contact, the body.

The experiments measure whether each HMC system correctly locates the joints in the labeled categories or not.

#### 5.2.2. Limb Length Adaptation

The limb lengths of the performers were also analyzed in the second experiment. Table 3 shows the differences between limb length values obtained after the learning phase and real limb length values. Despite the noisy pose estimation the OpenNI provides for the hands, forearm mean errors are below 3.5 cm. On the other hand, the mean estimation errors for the arm lengths are below 0.5 cm.

#### 5.2.3. Evaluation of Pose Estimation Results for Occluded and Overlapped Joints

The second set of experiments provided about 25 min of labeled motion data. Table 4 shows the results provided by the OpenNI tracker and the proposed model-based HMC system. Joint positions are successfully estimated for a frame if they are inside its labeled category.

Results in Table 4 show that the motion of hands and elbows around the head is robustly estimated by the OpenNI tracker, even if occlusions and overlapping is present. The elbow positions, particularly, are correctly inferred for nearly all the frames. The use of a model to reinforce the OpenNI data improves these results, but only marginally.

**Figure 7.**
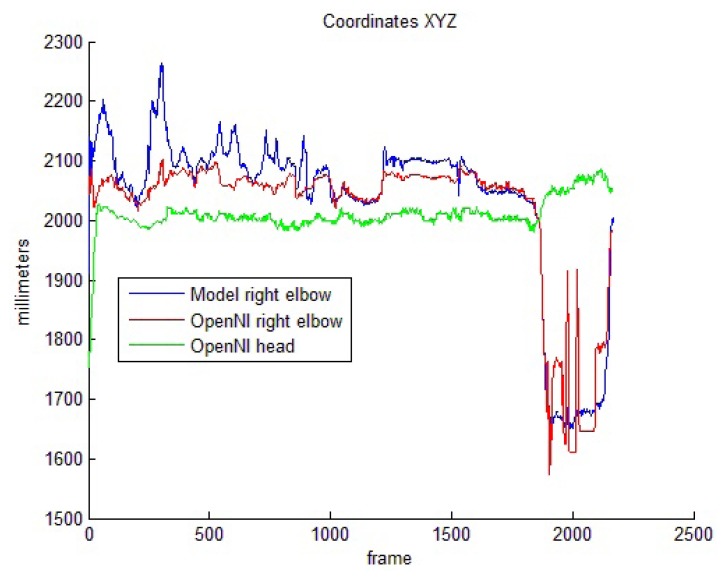
Camera-joint distances for a head-related test sequence.

Figure 7 shows the camera-joint distances for one of these head-related sequences. As demonstrated in Section 5.1, the model-based reinforcement offers a worse estimation for the elbow positions until arm lengths adapt to real values. Once the learning phase is complete, the elbow follows a nearly similar trajectory for both the OpenNI tracker and the proposed system. This trajectory becomes noisier for the OpenNI tracker between frames 1,800 and 2,200, in which the elbows move closer to the camera and overlap with the shoulders and the head. These issues, however, do not prevent the tracker from correctly identifying the pose of the joint.

**Figure 8.**
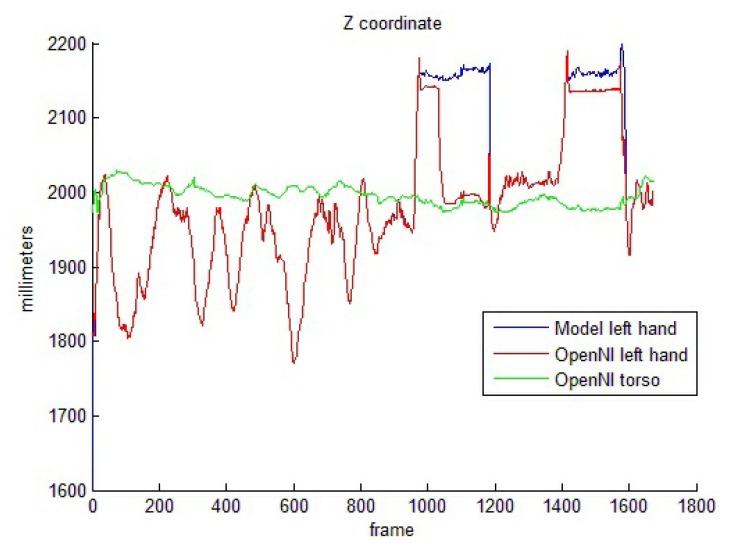
Camera-joint distances for a torso-related test sequence.

Torso-related motion offers a completely different set of results. The performance of the OpenNI tracker decays if the hands move near the torso. Table 4 shows success percentages of around 50% for both hand and elbow pose estimations. The model-based pose reinforcement, in these cases, improves the estimation results significantly, as depicted. In order to better understand these improvements, Figure 8 shows the camera-joint distance for one of the test sequences, in which the performer hides her left hand behind the body two times. The OpenNI tracker usually sets the depth value for the hidden joint to a too close value, with respect to the visible joint (*i.e.*, in frames 1,100–1,200, the hand and the body are overlapped). The model avoids these odd poses and maintains the joints in valid positions.

These tests show that the model-based reinforcement significantly improves OpenNI pose estimations, regarding hidden or overlapping joints. The computational cost of the model-based kinematics filter, on the other hand, is low. The complete reinforcement process takes an average of three milliseconds on a standard PC. Besides, the two stages can be easily split to improve performance. In this paper, the OpenNI tracker and the model-based reinforcement stage are executed in parallel threads. This division allows the system to maintain a process speed of 30 frames per second (*i.e.*, the same speed as a system that includes only the OpenNI tracker).

## 6. Conclusions

The OpenNI tracker has been extensively employed in the last year. Recent contributions include different qualitative and quantitative evaluations of this tracker [9]. These tests show that the method is adequate for most applications that the Kinect system is designed for (*i.e.*, video games). However, these contributions do not analyze if the 3D centroids provided by the tracker correspond to a valid human pose or not.

The quantitative tests presented in this paper highlight one important issue of the OpenNI tracker: the perception of the hand centroid is noisy, due to the high variations the hand pose introduces in the depth map (e.g., the hand centroid will move several centimeters if a fist changes to an open hand). The model-based pose refinement phase is not able to compensate for these errors.

The benefits of the proposed system are shown by the elbow pose estimation results in Experiment 1 and, specially, the pose estimations in motion involving occluded or overlapped joints (Experiment 2). The model-based pose refinement phase improves the accuracy of the OpenNI tracker, reducing the errors introduced by perception noise, ambiguities and occlusions. It also guarantees that the final provided pose corresponds to a valid human body configuration. The pose refinement phase last an average of 3 ms per frame and can be executed in parallel to the OpenNI tracker, allowing the whole HMC process to run at 30 fps.

Learning the proportions of the human performer is an important feature of the proposed system. Results show that a model using unadjusted limb lengths may not be adequate to reinforce the OpenNI tracker results, as it may increase final position errors. This paper proposes a limb length learning algorithm able to converge towards precise and stable values for performers of different heights, gender and proportions. The results included in the paper show that such an adapted model is able to significantly improve the results of the OpenNI tracker.

The work described in this paper will continue through research conducted in the next months. First of all, the use of a more complex model will be evaluated, especially regarding the shoulder complex. Tests involving leg pose refinement will be also conducted. The use of the proposed model to reinforce the Microsoft Kinect SDK tracker will also be addressed. Finally, the use of a model-based reinforcement phase may help in detecting and successfully tracking movements in which the person interacts with objects or with other people. Thus, dynamic changes in the kinematic chain and interactions between different models will be addressed in future works.

Following previous works [2], the current research of the authors points towards improving the perceptive, interactive and learning capabilities of robots working in daily life environments or in more specific rehabilitation scenarios [26,27]. The HMC system proposed in this paper suits the requirements of these applications and will be incorporated into the perceptual modules of the used robots.

## Figures and Tables

**Table 1.** Mean errors and standard deviations of right hand joints, in centimeters.

	**Hand**		**Elbow**		**Shoulder**	

	**Mean err**	**SD**	**Mean error**	**SD**	**Mean error**	**SD**
OpenNI centroids	11.3 cm	5.3 cm	6.7 cm	2.7 cm	2.4 cm	1.0 cm
Fixed limb lengths	11.7 cm	5.0 cm	7.2 cm	2.3 cm	2.5 cm	1.0 cm
Adaptive limb lengths	11.7 cm	5.0 cm	4.9 cm	1.7 cm	2.5 cm	1.0 cm

**Table 2.** Motion sequences employed in Experiment 2.

**Test N^o^**	**Ref.Joint**	**Θ***_d_* **(mm)**	**Brief Description**
#1	Head	50	Hands touch the nape. Elbows move covering the face.
#2	Head	50	Hands alternatively touch the nape and the nose.
#3	Torso	100	Hands move behind the torso.
#4	Torso	200	Torso rotates left and right.

**Table 3.** Limb length errors in Experiment 2.

**Limb**	**Mean Error (cm)**	**SD (cm)**
Upper arm	0.42	0.37
Forearm	3.3	1.97

**Table 4.** Percentages of correctly estimated hands and elbows poses.

	**Hands**		

	**Evaluated Frames**	**OpenNI Tracker**	**Proposed Algorithm**
Total	36,810	58.47%	74.90%
Head-related	18,759	67.56%	69.19%
Torso-related	18,051	49.03%	80.82%

			
	**Elbows**		

	**Evaluated Frames**	**OpenNI Tracker**	**Proposed Algorithm**

Total	29,129	68.34%	85.32%

Head-related	12,070	92.37%	96.44%
Torso-related	17,059	51.35%	77.45%
